# Variability in Survival Outcomes Among Asian Ethnic Groups with Stage IV NSCLC

**DOI:** 10.3390/medicina61040753

**Published:** 2025-04-19

**Authors:** Aria Bassiri, Yue-Lin Hu, Christina Boutros, Boxiang Jiang, Jillian Sinopoli, Leonidas Tapias Vargas, Philip A. Linden, Christopher W. Towe

**Affiliations:** 1 Division of Thoracic and Esophageal Surgery, Department of Surgery, University Hospitals Cleveland Medical Center, 11100 Euclid Avenue, Cleveland, OH 44106, USA; aria.bassiri@uhhospitals.org (A.B.); christina.boutros@uhhospitals.org (C.B.); boxiang.jiang@uhhospitals.org (B.J.); jillian.sinopoli@uhhospitals.org (J.S.); leonidas.tapias@uhhospitals.org (L.T.V.); philip.linden@uhhospitals.org (P.A.L.); 2 School of Medicine, Case Western Reserve University, 9501 Euclid Avenue, Cleveland, OH 44106, USA; yxh1365@case.edu

**Keywords:** lung cancer, Asian, race, survival, stage IV

## Abstract

*Background and Objectives*: Patients of Asian descent are often grouped together despite their diverse ethnicities and genetic backgrounds. Cancer outcomes result from a complex interplay of genetics, environment, and socioeconomic factors. This study aims to describe lung cancer survival outcome variations in Asian ethnic subgroups, hypothesizing that significant outcome differences exist between subgroups. *Materials and Methods*: A retrospective analysis of the 2020 National Cancer Database identified patients with stage IV non-small-cell lung cancer (NSCLC). Asian patients were subcategorized into nine groups: Chinese, Japanese, Korean, Asian Indian/Pakistani, Vietnamese, Pacific Islander, Filipino, Laotian/Hmong/Kampuchean/Thai, and Other Asian/Asian not otherwise specified (NOS). The primary outcome was overall survival, and the secondary outcome was utilization of palliative care. Kaplan–Meier analysis and multivariate Cox and logistic modeling were used to assess outcomes of interest. *Results*: A total of 23,747 Asian patients with stage IV NSCLC were identified. Demographic characteristics of the subgroups varied by age, sex, Charlson–Deyo Comorbidity Index, and utilization of palliative care. Relative to Chinese Asians, multivariate Cox analysis showed worse survival outcomes among patients categorized as Japanese, Korean, Pacific Islanders, Filipino, and Laotian/Hmong/Kampuchean/Thai. The rate of palliative care utilization also varied among Asian subgroups. Compared to Chinese patients, palliative care was more likely to be utilized by patients categorized as Japanese and Pacific Islander. *Conclusions*: Amongst Asian subgroups, variations in survival outcomes and palliative care utilization in stage IV NSCLC patients were observed. Surgeons should acknowledge these disparities and consider disaggregating Asian races in prognosis analysis to enhance understanding of race’s impact on outcomes. Recognizing these differences is crucial for guiding personalized treatment strategies, optimizing resource allocation, and informing health policy to ensure equitable cancer care for all Asian populations.

## 1. Introduction

Lung cancer is responsible for the highest number of cancer-related fatalities worldwide (18.4%) [1]. The variances in incidence, progression, and survival outcomes of many cancers among different racial and ethnic groups suggest a complex interplay of genetic, environmental, and socioeconomic factors [2,3,4,5]. Specifically, significant disparities have been noted in the rates and outcomes of cancer among distinct Asian subgroups, despite their often-misleading categorization as a homogeneous group in cancer research [6,7]. Consequently, it is crucial to move beyond this oversimplification and gain a comprehensive understanding of the influence of race on lung cancer prognosis [8]. This study aims to address this issue by analyzing the overall survival outcomes of late-stage lung cancer patients while considering disaggregated Asian races.

Previous research has revealed that lung cancer exhibits considerable genetic heterogeneity, characterized by distinct gene expression patterns, somatic mutation profiles, and susceptibility to specific genetic alterations [4,9]. Recent advancements in genomics have further highlighted the existence of ethnicity-specific genetic variants associated with lung cancer [10]. Lumping together individuals of Asian descent as a single group overlooks these intricate genetic differences and their potential impacts on survival outcomes. For instance, East Asians demonstrate a higher prevalence of epidermal growth factor receptor (EGFR) missense and deletion mutations, which can have significant implications for prognosis and treatment response. This is due to lung cancer’s predilection for amplification of the EGFR gene, as well as the fact that several treatments for NSCLC inhibit EGFR [11,12,13]. However, this crucial information may be diluted when considering Asians as a whole, thus obscuring the true influence of these genetic variations on patient outcomes. Focusing on Stage IV is clinically significant, as it has poor survival rates and a heavy reliance on systemic therapies like targeted treatments and biomarker-driven therapies (EGFR, ALK, PD-L1). Given that Asian populations have a higher prevalence of EGFR mutations, disaggregating data can refine treatment approaches.

The purpose of this study is to utilize data from the National Cancer Database to discern disparities in overall survival outcomes among late-stage lung cancer patients from diverse Asian subgroups. We hypothesize that different Asian subgroups will exhibit varying rates of survival in lung cancer.

## 2. Materials and Methods

### 2.1. Data Source

A retrospective cohort study was performed using the National Cancer Database (NCDB). The NCDB is a joint program of the Commission on Cancer (CoC) of the American College of Surgeons (ACS) and the American Cancer Society. This is a nationwide database containing de-identified oncologic outcomes from more than 1500 commissioned, accredited cancer programs in the USA and Puerto Rico. It is estimated that this database captures nearly 70% of all newly diagnosed cancer. ACS and CoC have not verified and are not responsible for the statistical validity of the data analysis or the conclusions derived by investigators. Definitions of database variables are available from the NCDB Participant User Data File data dictionary [14].

### 2.2. Patient Population

**Figure 1:**
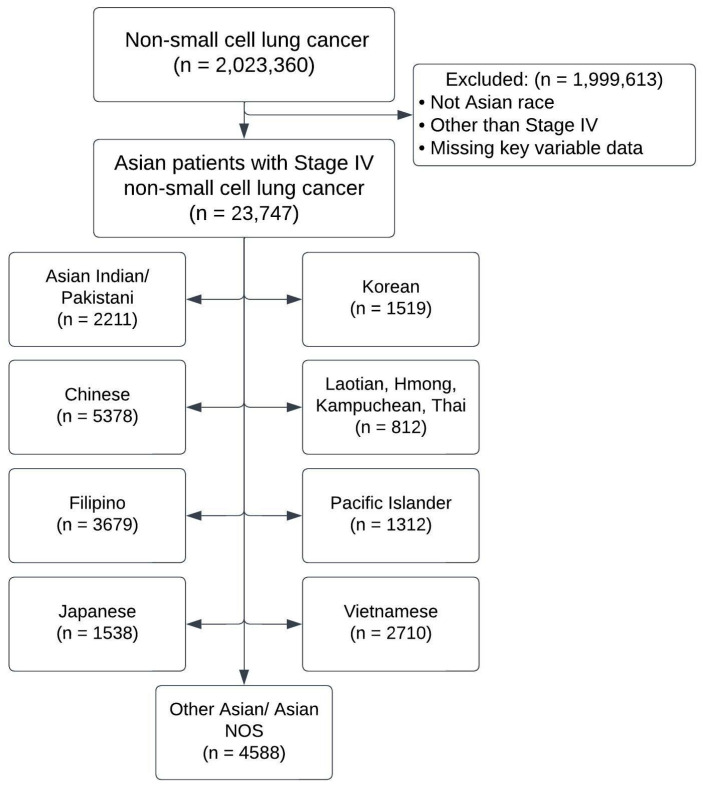
Retrospective database analysis of the 2020 National Cancer Database of Asian patients with Stage IV non-metastatic, non-small-cell lung cancer. Distribution of Asian subgroup among the larger cohort of Asian patients. NOS—not otherwise specified.

We identified patients with clinical Stage IV non-small-cell lung cancer from 2004 to 2020 in the NCDB. Patients were categorized as Asian patients and subcategorized into nine groups based on the “race” data element: Chinese, Japanese, Korean, Asian Indian/Pakistani, Vietnamese, Pacific Islander, Filipino, Laotian/Hmong/Kampuchean/Thai, and Other Asian/Asian not otherwise specified (NOS). Patients were excluded if classified as not Asian, if they had other than Stage IV disease, or if they were missing key data variables (Figure 1). The study design was created using a Strengthening the Reporting of Observational Studies in Epidemiology (STROBE) checklist, which included guidelines on an accurate and complete report of an observational study [15].

### 2.3. Outcome Measure

The primary outcome of interest was overall survival, and the secondary outcome was utilization of palliative care

### 2.4. Statistical Analysis

Variables included in the analysis were age, sex, race, Charlson–Deyo (CD) comorbidities, type of facility, tumor histology, and palliative care use. Demographic, clinical, and institutional characteristics were compared among race subgroups. Statistical comparison among the groups was performed using the Wilcoxon rank sum test for continuous variables and Chi-square for categorical variables. Demographics and clinical characteristics are described using median and interquartile range (IQR) for continuous variables and frequency and percentages for categorical variables. All continuous variables were assumed to be non-normally distributed, and all analyses of continuous variables were conducted accordingly. The multivariate Cox regression adjusted for pertinent variables, and a Kaplan–Meier (KM) analysis and a log–rank test were performed to assess for overall survival. The likelihood of palliative care utilization (surgery, radiation, chemotherapy, immunotherapy, and pain management) among the Asian subgroups was evaluated with multivariate logistic regression. Statistical analysis was performed using STATA MP (Version 17.0, College Station, TX, USA). Statistical significance was defined as *p*-value ≤ 0.05. This study was determined to be exempt from Institutional Review Board review and did not require informed consent for the use of deidentified data.

### 2.5. Method Limitations

This study is subject to potential biases from unmeasured confounders, such as socioeconomic status, comorbidities, access to specialized oncology care, and cultural factors influencing treatment decisions. Additionally, the retrospective nature of the dataset may introduce selection bias, and the reliance on broad population-based registries may not fully capture granular clinical details, such as molecular biomarker status and treatment adherence. These limitations should be considered when interpreting the findings, and future studies incorporating prospective data and more detailed clinical variables are warranted.

## 3. Results

### 3.1. Asian Subgroup Distribution in Stage IV NSCLC

**Figure 2:**
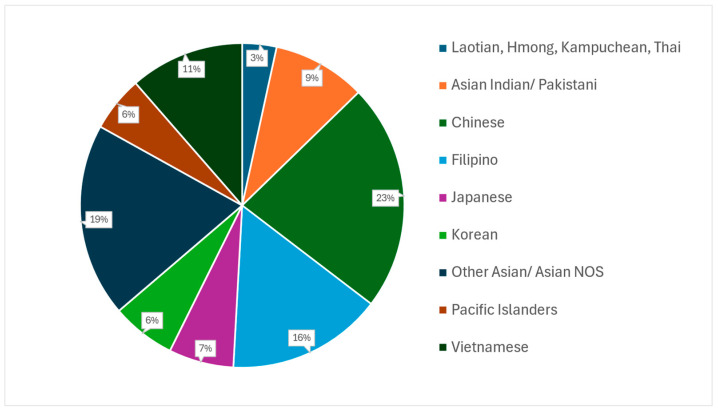
Distribution of Asian subgroups with Stage IV non-metastatic, non-small-cell lung cancer from 2004 to 2020 in the National Cancer Database. NOS—not otherwise specified.

A total of 23,747 patients who were classified as being of Asian race with stage IV NSCLC were identified. The distribution of the subgroup of Asian races is depicted in Figure 2. The most common subgroup was Chinese (5378, 22.7%), followed by Other Asian/Asian NOS (4588, 19.3%), Filipino (3679, 15.5%), Vietnamese (2710, 11.4%), Asian Indian/Pakistani (2211, 9.3%), Japanese (1538, 6.5%), Korean (1519, 6.4%), Pacific Islander (1312, 5.5%), and Laotian/Hmong/Kampuchean/Thai (812, 3.4%). Demographic and clinical characteristics were compared among the different subgroups using univariate analyses (Table 1).

### 3.2. Survival Disparities Among Asian Subgroups in Unadjusted Cox Analysis

In an unadjusted Cox analysis (Table 2), significant differences in overall survival were observed among Asian subgroups. Compared with Chinese patients, the Filipino (HR: 1.28, 95% CI: 1.18–1.39, *p* < 0.001), Japanese (HR: 1.73, 95% CI: 1.55–1.93, *p* < 0.001), Korean (HR: 1.16, 95% CI: 1.05–1.28, *p* = 0.003), Laotian/Hmong/Kampuchean/Thai (HR: 1.39, 95% CI: 1.23–1.58, *p* < 0.001), and Pacific Islander (HR: 1.54, 95% CI: 1.37–1.74, *p* < 0.001) groups had significantly worse survival outcomes. Vietnamese patients trended toward worse survival outcomes compared with Chinese patients, but this was not statistically significant (HR: 1.11, 95% CI: 1.00–1.23, *p* = 0.051). Asian Indian/Pakistani (HR: 0.97, 95% CI: 0.86–1.58, *p* = 0.622) and Other Asian/Asian Not Otherwise Specified (NOS) (HR: 1.02, 95% CI: 0.94–1.11, *p* = 0.694) groups demonstrated no significant difference in survival outcomes relative to the Chinese subgroup.

Median survival also varied among ethnic subgroups. The Chinese subgroup had the longest median survival (14.85 months, 95% CI: 13.90–15.70), whereas the Japanese (6.01 months, 95% CI: 5.52–6.80), Pacific Islander (6.54 months, 95% CI: 5.88–7.69), and Laotian/Hmong/Kampuchean/Thai (7.49 months, 95% CI: 6.51–8.90) subgroups had the shortest median survival. The Filipino (9.63 months, 95% CI: 8.67–10.38) and Korean (11.66 months, 95% CI: 10.05–13.04) groups also demonstrated lower median survival compared with the reference group.

Five-year survival estimates followed a similar trend, with the highest survival rates seen in the Asian Indian/Pakistani group (19.91%, 95% CI: 17.81–22.08) and the lowest in the Japanese subgroup (5.25%, 95% CI: 3.03–6.69). The Filipino (11.31%, 95% CI: 10.08–12.61), Laotian/Hmong/Kampuchean/Thai (12.61%, 95% CI: 10.06–15.46), and Pacific Islander (7.61%, 95% CI: 5.98–9.50) groups also exhibited poor long-term survival outcomes.

### 3.3. Race and Survival

**Figure 3:**
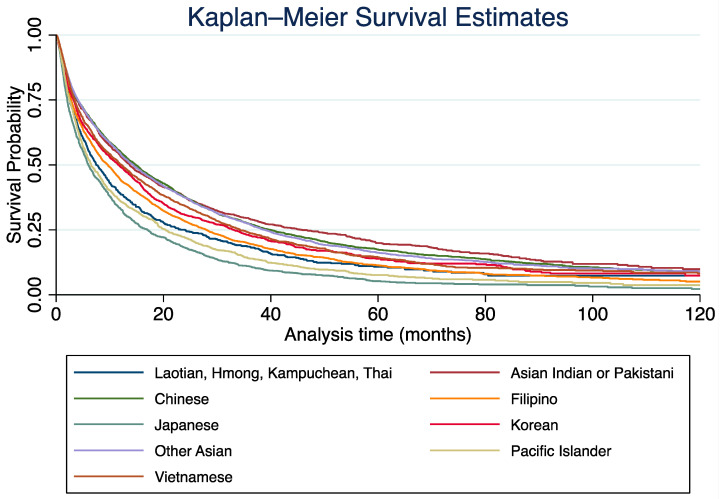
Kaplan–Meier survival analysis among different subgroups of Asian patients with stage IV non-small-cell lung cancer from 2004 to 2020 in the National Cancer Database.

In the multivariate adjusted Cox regression (Table 3), significant disparities in survival outcomes were observed among Asian subgroups as compared with the Chinese reference group. The Filipino (HR: 1.21, 95% CI: 1.12–1.31, *p* < 0.001), Japanese (HR: 1.47, 95% CI: 1.36–1.60, *p* < 0.001), Korean (HR: 1.11, 95% CI: 1.01–1.22, *p* = 0.034), Laotian/Hmong/Kampuchean/Thai (HR: 1.48, 95% CI: 1.31–1.67, *p* < 0.001), and Pacific Islander (HR: 1.46, 95% CI: 1.26–1.69, *p* < 0.001) groups exhibited significantly worse survival outcomes. The Vietnamese subgroup demonstrated a trend toward worse survival outcomes (HR: 1.07, 95% CI: 0.98–1.18), but this was not statistically significant (*p* = 0.112). The Asian Indian/Pakistani (HR: 0.96, 95% CI: 0.87–1.06, *p* = 0.436) and Other Asian/Asian NOS (HR: 1.03, 95% CI: 0.96–1.11, *p* = 0.356) groups did not show significant differences in survival outcomes relative to the Chinese group. Differences in survival outcomes were further demonstrated by the KM analysis and log–rank test (Figure 3 and Table 2. Log–rank test: *p* < 0.001).

### 3.4. Demographic and Clinical Factors

Increasing age was associated with poorer survival outcomes (HR: 1.02, 95% CI: 1.02–1.02, *p* < 0.001). Male sex was also a significant predictor of worse survival outcomes (HR: 1.28, 95% CI: 1.23–1.33, *p* < 0.001) compared with the female sex.

### 3.5. Histology and Survival

Histologic subtype was strongly associated with survival outcomes. Compared with adenocarcinoma, patients with squamous cell carcinoma (HR: 1.36, 95% CI: 1.29–1.43, *p* < 0.001) and other histologic subtypes (HR: 1.37, 95% CI: 1.31–1.43, *p* < 0.001) had significantly worse survival outcomes.

### 3.6. Comparison of Survival Between Asian and Non-Asian Patients

Asian patients with stage IV NSCLC exhibited significantly better survival outcomes compared with non-Asian patients. In an unadjusted analysis, the median survival time for Asian patients was 11.5 months (95% CI: 11.2–11.9) versus 5.5 months (95% CI: 5.5–5.6) for non-Asian patients (log–rank test *p* < 0.001). Multivariable Cox regression confirmed superior survival outcomes among Asian patients relative to non-Asian patients (HR 0.70; 95% CI: 0.67–0.74; *p* < 0.001), after adjusting for age, sex, histology, comorbidity score, and treatment facility type.

### 3.7. Race and Palliative Care Utilization

Compared with the Chinese reference group, significant differences in palliative care utilization were observed among certain Asian subgroups (Table 4). Japanese patients were significantly more likely to utilize palliative care (OR: 1.58, 95% CI: 1.08–2.48, *p* = 0.046). Similarly, Pacific Islander patients had increased odds of palliative care use (OR: 1.76, 95% CI: 1.07–2.90, *p* = 0.025).

In contrast, other Asian subgroups, including Asian Indian/Pakistani (OR: 1.18, 95% CI: 0.90–1.53, *p* = 0.228), Filipino (OR: 1.03, 95% CI: 0.73–1.44, *p* = 0.880), Korean (OR: 1.09, 95% CI: 0.86–1.39, *p* = 0.479), Laotian/Hmong/Kampuchean/Thai (OR: 1.13, 95% CI: 0.81–1.58, *p* = 0.454), Vietnamese (OR: 1.04, 95% CI: 0.82–1.32, *p* = 0.721), and Other Asian/Asian NOS (OR: 0.98, 95% CI: 0.78–1.23, *p* = 0.849) did not show significant differences in palliative care utilization compared with the Chinese group.

## 4. Discussion

This is a retrospective analysis utilizing a national database, evaluating survival outcomes among Asian patients with stage IV NSCLC. Our analysis demonstrates variations in survival outcomes and palliative care utilization in stage IV NSCLC patients among Asian subgroups. These findings underscore the importance of disaggregating data by specific Asian ethnicities rather than treating them as a homogeneous group, as is traditionally done in many cancer studies. By doing so, our study reveals nuanced differences that are crucial for understanding and addressing health disparities in lung cancer prognosis.

The consideration of race in medical research prompts us to explore the role of genetic and sociocultural factors in health disparities. While the founder effect theory posits that distinct racial groups may exhibit unique genetic variations, studies consistently demonstrate a greater extent of genetic variation within racial groups compared with variations among them [16,17]. However, it is crucial to recognize that race remains a critical factor in certain diseases, particularly in the realm of cancer research. In the case of lung cancer, the association of EGFR mutations within Asian races has been extensively documented [18]. This observation aligns with the principles of the founder effect, as certain Asian subgroups may have experienced isolation and subsequent genetic drift, resulting in a higher prevalence of EGFR mutations. However, it is important to note that this association may primarily manifest in specific subsets of Asian races, rather than encompassing the entire Asian population.

In some studies, Asian race is often aggregated as a whole, overlooking the inherent diversity and genetic variations that exist within this broad racial category [7,18,19,20]. This practice fails to recognize the intricate interplay of genetic, environmental, and sociocultural factors that contribute to disparities in lung cancer outcomes among different Asian subgroups [5,21,22]. By treating Asians as a monolithic group, the nuanced differences in genetic profiles, prevalence of specific mutations like EGFR, and sociodemographic characteristics are overshadowed, limiting our understanding of the unique challenges and potential targeted interventions needed for each subgroup [7,21,22]. In addition to genetic factors, environmental and sociocultural elements play critical roles in cancer outcomes. Access to healthcare, socioeconomic status, cultural attitudes toward medical treatment, and lifestyle choices are all influential. For example, differences in smoking rates, dietary habits, and exposure to environmental pollutants can vary significantly among Asian subgroups, impacting lung cancer incidence and progression. Our study’s findings that Filipino, Japanese, Korean, Laotian/Hmong/Kampuchean/Thai, and Pacific Islander subgroups have inferior survival outcomes compared with Chinese patients highlight the potential influences of these non-genetic factors. Palliative care utilization also varied among the subgroups, with Japanese and Pacific Islanders being more likely to receive palliative care compared with Chinese patients. This could reflect differences in healthcare access, cultural acceptance of palliative care, or the availability of resources.

Understanding these sociocultural differences is crucial for developing targeted interventions to improve cancer care equity, and disaggregating Asian data in national cancer registries and clinical trials would allow for more precise public health strategies and guideline development. This would amend the need for better representation of Asian subgroups in clinical trials. Future trial designs should ensure adequate subgroup enrollment to assess differential treatment responses. Additionally, health societies should consider race/ethnicity-specific recommendations in lung cancer screening, biomarker testing, and treatment guidelines to improve outcomes across diverse populations. Moreover, differences in palliative care utilization suggest potential disparities in healthcare access, patient preferences, or provider biases. Targeted efforts to improve palliative and supportive care access for underutilized subgroups could enhance quality of life and end-of-life care.

The significant variations in lung cancer outcomes among Asian subgroups have important implications for clinical practice and research. Clinicians should be aware of these differences and consider them when planning treatment strategies. Personalized medicine approaches, which tailor treatment based on genetic and demographic factors, could be particularly beneficial. For instance, targeted therapies for patients with EGFR mutations could be prioritized for those subgroups with higher mutation prevalence. For researchers, our study underscores the need for more granular data analysis. Future studies should strive to collect and analyze data at the subgroup level to uncover hidden disparities and inform more equitable healthcare practices. Additionally, prospective studies are needed to validate our findings and explore causal relationships among race, genetic mutations, and survival outcomes.

### Limitations

Several limitations should be acknowledged in this study. First, the analysis of Asian subgroups was based on available data from the NCDB, which relies on hospital registry data and may not capture the full extent of genetic, environmental, and sociocultural heterogeneity within the Asian subgroups. Second, the retrospective nature of the study design limits our ability to establish causal relationships between race and lung cancer outcomes. Other unmeasured confounding factors could potentially influence the observed associations. Third, the generalizability of our findings may be limited to the population covered by the NCDB, which represents a subset of cancer cases and may not fully represent the broader population. Finally, the study focused primarily on survival outcomes and did not explore other important aspects, such as treatment response or quality of life. Future research should address these limitations by incorporating larger and more diverse datasets, considering prospective study designs, and exploring additional factors that contribute to disparities among Asian subgroups.

## 5. Conclusions

Among the Asian subgroups, there are variations in survival outcomes and palliative care utilization in stage IV non-small-cell lung cancer. Disaggregating the Asian races in the analysis of lung cancer prognosis is essential for gaining a more comprehensive understanding of the impact of race and ethnicity on patient outcomes. By recognizing the genetic, environmental, and sociocultural variations within Asian subgroups, we can uncover previously obscured correlations and tailor treatment strategies accordingly. Prospective studies integrating precision medicine approaches will be essential to ensure that racial and ethnic disparities do not persist in the era of targeted therapies and personalized oncology.

## Figures and Tables

**Table 1:** Demographic and clinical factors associated with Asian patients with Stage IV non-small cell lung cancer from 2004 to 2020 in the National Cancer Database. IQR—interquartile range. NOS—not otherwise specified.

	Asian Indian/Pakistani	Chinese	Filipino	Japanese	Korean	Laotian, Hmong, Kampuchean, Thai	Pacific Islander	Vietnamese	Other Asian/Asian NOS	*p*-Value
Age	Mean: 64.5 Median: 65 (IQR 56–74)	Mean: 67.5 Median: 68 (IQR 60–76)	Mean: 67.2 Median: 68 (IQR 60–76)	Mean: 73.5 Median: 75 (IQR 67–82)	Mean: 67.8 Median: 69 (IQR 60–77)	Mean: 62.9 Median: 63 (IQR 56–71)	Mean: 64.9 Median: 65 (IQR 58–73)	Mean: 64.3 Median: 64 (IQR 56–74)	Mean: 65.4 Median: 66 (IQR 57–75)	<0.001
Sex										<0.001
Male	1348 (61.0%)	2821 (52.5%)	1925 (52.3%)	708 (46.0%)	766 (50.4%)	441 (54.3%)	702 (53.5%)	1614 (59.6%)	2246 (49.0%)	
Female	863 (39.0%)	2557 (47.5%)	1754 (47.7%)	830 (54.0%)	753 (49.6%)	371 (45.7%)	610 (46.5%)	1096 (40.4%)	2342 (51.0%)	
Chalrson-Deyo Comorbidities										<0.001
0	1580 (71.5%)	4184 (77.8%)	2557 (69.5%)	1086 (70.6%)	1121 (73.8%)	621 (76.5%)	822 (62.6%)	2043 (75.4%)	3511 (76.5%)	
1	433 (19.6%)	836 (15.5%)	729 (19.8%)	288 (18.7%)	291 (19.2%)	138 (76.5%)	276 (21.0%)	467 (17.2%)	728 (15.9%)	
2	113 (5.1%)	212 (3.9%)	243 (6.6%)	110 (8.9%)	71 (4.7%)	138 (3.3%)	116 (8.8%)	124 (10.0%)	224 (4.9%)	
3+	85 (3.8%)	146 (2.7%)	150 (4.1%)	54 (6.8%)	36 (2.4%)	24 (1.9%)	98 (7.5%)	76 (2.8%)	125 (2.7%)	
Facility type										<0.001
Community cancer program	129 (6.1%)	238 (4.5%)	449 (12.4%)	232 (15.2%)	65 (4.3%)	48 (6.1%)	128 (9.9%)	166 (6.3%)	250 (5.6%)	
Comprehensive community program	669 (31.4%)	1448 (27.5%)	1083 (30.0%)	463 (30.3%)	442 (29.4%)	267 (34.1%)	352 (27.3%)	1130 (42.7%)	352 (27.3%)	
Academic/research program	991 (46.5%)	2961 (56.2%)	1307 (36.2%)	529 (34.6%)	724 (48.2%)	302 (38.5%)	538 (41.7%)	847 (32.0%)	538 (41.7%)	
Integrated network cancer program	341 (16.0%)	621 (11.8%)	775 (21.4%)	304 (19.9%)	271 (18.0%)	167 (21.3%)	272 (21.1%)	504 (19.0%)	272 (21.1%)	
Histology group										<0.001
Adenocarcinoma	1650 (74.6%)	4080 (75.9%)	2757 (74.9%)	986 (64.1%)	1047 (68.9%)	595 (73.3%)	896 (68.3%)	2091 (77.2%)	3519 (76.7%)	
Squamous	233 (10.5%)	558 (10.4%)	390 (10.6%)	248 (16.1%)	226 (14.9%)	87 (10.7%)	220 (16.8%)	226 (8.3%)	462 (10.1%)	
other	328 (14.8%)	740 (13.8%)	532 (14.5%)	304 (19.8%)	246 (16.2%)	130 (16.0%)	196 (14.9%)	393 (14.5%)	607 (13.2%)	
Palliative care										
No	1783 (80.6%)	4452 (82.8%)	3055 (83.0%)	1187 (77.2%)	1239 (81.6%)	660 (81.3%)	956 (72.9%)	2257 (83.3%)	3841 (83.7%)	
Yes	428 (19.4%)	926 (17.2%)	624 (17.0%)	351 (22.8%)	280 (18.4%)	152 (18.7%)	356 (27.1%)	453 (16.7%)	747 (16.3%)	

**Table 2:** Unadjusted Cox analysis and Kaplan–Meier survival analysis (median survival and 5-year survival) among Asian patient with Stage IV non-small-cell lung cancer from 2004 to 2020 in the National Cancer Database. CI—confidence interval. NOS—not otherwise specified.

Race	Hazard Ratio (95% Confidence Interval, *p*-Value)	Median Survival in Months (95% Confidence Interval)	5-Year Overall Survival (95% Confidence Interval)
Asian Indian/Pakistani	0.97 (0.86–1.58, 0.622)	13.70 (12.58–14.82)	19.91 (17.81–22.08)
Chinese	reference	14.85 (13.90–15.70)	17.37 (16.11–18.66)
Filipino	1.28 (1.18–1.39, <0.001)	9.63 (8.67–10.38)	11.31 (10.08–12.61)
Japanese	1.73 (1.55–1.93, <0.001)	6.01 (5.52–6.80)	5.25 (3.03–6.69)
Korean	1.16 (1.05–1.28, 0.003)	11.66 (10.05–13.04)	13.83 (11.74–16.09)
Laotian, Hmong, Kampuchean, Thai	1.39 (1.23–1.58, <0.001)	7.49 (6.51–8.9)	12.61 (10.06–15.46)
Pacific Islander	1.54 (1.37–1.74, <0.001)	6.54 (5.88–7.69)	7.61 (5.98–9.50)
Vietnamese	1.11 (1.00–1.23, 0.051)	12.16 (11.17–13.21)	14.45 (12.85–16.14)
Other Asian/Asian NOS	1.02 (0.94–1.11, 0.694)	14.29 (13.40–15.24)	16.13 (14.76–17.55)

**Table 3:** Multivariate Cox regression adjusting for patient characteristics of factors associated with survival among Asian patients with Stage IV non-small-cell lung cancer from 2004 to 2020 in the National Cancer Database. NOS—not otherwise specified.

	Hazard Ratio (95% Confidence Interval)	*p*-Value
Race		
Asian Indian/ Pakistani	0.96 (0.87–1.06)	0.436
Chinese	Reference	
Filipino	1.21 (1.12–1.31)	<0.001
Japanese	1.47 (1.36–1.60)	<0.001
Korean	1.11 (1.01–1.22)	0.034
Laotian, Hmong, Kampuchean, Thai	1.48 (1.31–1.67)	<0.001
Pacific Islander	1.46 (1.26–1.69)	<0.001
Vietnamese	1.07 (0.98–1.18)	0.112
Other Asian/Asian NOS	1.03 (0.96–1.11)	0.356
Age	1.02 (1.02–1.02)	<0.001
Sex		
Male	1.28 (1.23–1.33)	<0.001
Female	Reference	
Charlson–Deyo Comorbidities		
0	Reference	
1	1.16 (1.11–1.20)	<0.001
2	1.41 (1.29–1.55)	<0.001
3+	1.60 (1.41–1.81)	<0.001
Facility type		
Community cancer program	Reference	
Comprehensive community program	0.98 (0.89–1.08)	0.654
Academic/research program	0.84 (0.74–0.95)	0.007
Integrated network cancer program	1.01 (0.92–1.12)	0.783
Histology group		
Adenocarcinoma	Reference	
Squamous	1.36 (1.29–1.43)	<0.001
other	1.37 (1.31–1.43)	<0.001

**Table 4:** Multivariate logistic regression adjusting for patient characteristics of factors associated with palliative care use among Asian patients with Stage IV non-small-cell lung cancer from 2004 to 2020 in the National Cancer Database. NOS—not otherwise specified.

	Odds Ratio (95% Confidence Interval)	*p*-Value
Race		
Asian Indian/Pakistani	1.18 (0.90–1.53)	0.228
Chinese	Reference	
Filipino	1.03 (0.73–1.44)	0.880
Japanese	1.58 (1.08–2.48)	0.046
Korean	1.09 (0.86–1.39)	0.479
Laotian, Hmong, Kampuchean, Thai	1.13 (0.81–1.58)	0.454
Pacific Islander	1.76 (1.07–2.90)	0.025
Vietnamese	1.04 (0.82–1.32)	0.721
Other Asian/Asian NOS	0.98 (0.78–1.23)	0.849
Age	0.99 (0.99–1.00)	0.002
Sex		
Male	1.00 (0.92–1.08)	0.959
Female	Reference	
Charlson–Deyo Comorbidities		
0	Reference	
1	1.21 (1.10–1.35)	<0.001
2	1.24 (1.05–1.48)	0.014
3+	1.48 (1.22–1.80)	<0.001
Facility type		
Community cancer program	Reference	
Comprehensive community program	0.87 (0.47–1.62)	0.653
Academic/research program	1.56 (0.72–3.38)	0.264
Integrated network cancer program	1.46 (0.77–2.75)	0264
Histology group		
Adenocarcinoma	Reference	
Squamous	1.04 (0.92–1.17)	0.538
other	0.94 (0.47–1.62)	0.653

## Data Availability

Data used in this study were collected from the National Cancer Database (NCDB) and are publicly available. The NCDB is a joint program of the Commission on Cancer (CoC) of the American College of Surgeons (ACS) and the American Cancer Society. Data access is to be processed through these entities.

## Abbreviations

The following abbreviations are used in this manuscript:

NSCLC	Non-small-cell lung cancer
NCDB	National Cancer Database
ASC	American College of Surgeons
EGFR	Epidermal growth factor receptor

## References

[1] Bray F., Ferlay J., Soerjomataram I., Siegel R.L., Torre L.A., Jemal A. (2018). Global cancer statistics 2018: GLOBOCAN estimates of incidence and mortality worldwide for 36 cancers in 185 countries. CA Cancer J. Clin..

[2] American Cancer Society (2024). Cancer Facts and Statistics. Cancer Facts & Statistics. https://www.cancer.org/research/cancer-facts-statistics/all-cancer-facts-figures/2024-cancer-facts-figures.html.

[3] Schabath M.B., Cote M.L. (2019). Cancer Progress and Priorities: Lung Cancer. Cancer Epidemiol. Biomark. Prev..

[4] Schabath M.B., Cress W.D., Muñoz-Antonia T. (2016). Racial and Ethnic Differences in the Epidemiology and Genomics of Lung Cancer. Cancer Control.

[5] Gomez S.L., Quach T., Horn-Ross P.L., Pham J.T., Cockburn M., Chang E.T., Keegan T.H.M., Glaser S.L., Clarke C.A. (2010). Hidden breast cancer disparities in Asian women: Disaggregating incidence rates by ethnicity and migrant status. Am. J. Public Health.

[6] Gomez S.L., Von Behren J., McKinley M., Clarke C.A., Shariff-Marco S., Cheng I., Reynolds P., Glaser S.L. (2017). Breast cancer in Asian Americans in California, 1988–2013: Increasing incidence trends and recent data on breast cancer subtypes. Breast Cancer Res. Treat..

[7] Bock S., Henley S.J., O’neil M.E., Singh S.D., Thompson T.D., Wu M. (2023). Cancer Distribution Among Asian, Native Hawaiian, and Pacific Islander Subgroups–United States, 2015–2019. Morb. Mortal. Wkly. Rep..

[8] Lee A.W., Mendoza R.A., Aman S., Hsu R., Liu L. (2022). Thyroid cancer incidence disparities among ethnic Asian American populations, 1990–2014. Ann. Epidemiol..

[9] Swanton C., McGranahan N., Starrett G.J., Harris R.S. (2015). APOBEC Enzymes: Mutagenic Fuel for Cancer Evolution and Heterogeneity. Cancer Discov..

[10] Shi H., Seegobin K., Heng F., Zhou K., Chen R., Qin H., Manochakian R., Zhao Y., Lou Y. (2022). Genomic landscape of lung adenocarcinomas in different races. Front. Oncol..

[11] Shigematsu H., Lin L., Takahashi T., Nomura M., Suzuki M., Wistuba I.I., Fong K.M., Lee H., Toyooka S., Shimizu N. (2005). Clinical and biological features associated with epidermal growth factor receptor gene mutations in lung cancers. J. Natl. Cancer Inst..

[12] Zhou W., Christiani D.C. (2011). East meets West: Ethnic differences in epidemiology and clinical behaviors of lung cancer between East Asians and Caucasians. Chin. J. Cancer.

[13] Liam C.-K., Pang Y.-K., Poh M.-E. (2014). EGFR mutations in Asian patients with advanced lung adenocarcinoma. J. Thorac. Oncol..

[14] National Cancer Data Base. https://www.facs.org/quality-programs/cancer-programs/national-cancer-database/puf/.

[15] von Elm E., Altman D.G., Egger M., Pocock S.J., Gøtzsche P.C., Vandenbroucke J.P., STROBE Initiative (2014). The Strengthening the Reporting of Observational Studies in Epidemiology (STROBE) Statement: Guidelines for reporting observational studies. Int. J. Surg..

[16] Raz D.J., Gomez S.L., Chang E.T., Kim J.Y., Keegan T.H., Pham J., Kukreja J., Hiatt R.A., Jablons D.M. (2008). Epidemiology of non-small cell lung cancer in Asian Americans: Incidence patterns among six subgroups by nativity. J. Thorac. Oncol..

[17] DeRouen M.C., Canchola A.J., Thompson C.A., Jin A., Nie S., Wong C., Lichtensztajn D., Allen L., Patel M.I., Daida Y.G. (2022). Incidence of Lung Cancer Among Never-Smoking Asian American, Native Hawaiian, and Pacific Islander Females. J. Natl. Cancer Inst..

[18] Rosell R., Moran T., Queralt C., Porta R., Cardenal F., Camps C., Majem M., Lopez-Vivanco G., Isla D., Provencio M. (2009). Screening for epidermal growth factor receptor mutations in lung cancer. N. Engl. J. Med..

[19] Narayan A.K., Chowdhry D.N., Fintelmann F.J., Little B.P., Shepard J.-A.O., Flores E.J. (2021). Racial and Ethnic Disparities in Lung Cancer Screening Eligibility. Radiology.

[20] Cranford H.M., Koru-Sengul T., Lopes G., Pinheiro P.S. (2023). Lung Cancer Incidence by Detailed Race–Ethnicity. Cancers.

[21] Lee R.J., Madan R.A., Kim J., Posadas E.M., Yu E.Y. (2021). Disparities in Cancer Care and the Asian American Population. Oncologist.

[22] Taparra K., Dee E.C., Dao D., Patel R., Santos P., Chino F. (2022). Disaggregation of Asian American and Pacific Islander Women With Stage 0-II Breast Cancer Unmasks Disparities in Survival and Surgery-to-Radiation Intervals: A National Cancer Database Analysis From 2004 to 2017. JCO Oncol. Pract..

